# Supplementary material for: RIG-I like receptor sensing of host RNAs facilitates the cell-intrinsic immune response to KSHV infection
Source: Nat Commun. 2018 Nov 19;9:4841. doi: 10.1038/s41467-018-07314-7 (PMC6242832; doi:10.1038/s41467-018-07314-7)
Supplement: Supplementary file 1 — Supplementary Information [file 41467_2018_7314_MOESM1_ESM.pdf]

Supplementary Information

RIG-I like receptor sensing of host RNAs facilitates the cell-intrinsic immune response to KSHV infection

Zhao et al.

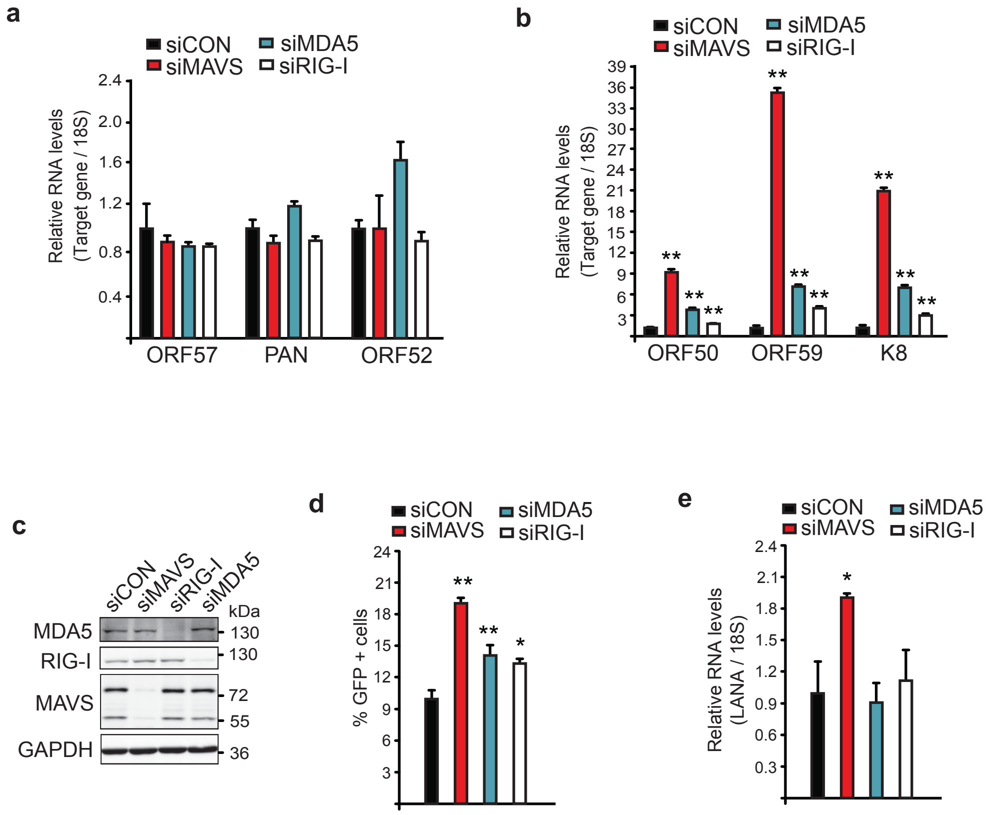

Supplementary Figure 1. Knockdown of RIG-I, MDA5, and MAVS enhances KSHV lytic reactivation in iSLK.219 cells but has a minimal effect on *de novo* infection. (a and b) iSLK.219 cells were transfected with indicated siRNAs for 48 h and then treated with or without Dox for 24 h. RNA was extracted from cells and expression of the indicated viral genes was quantified in latency (a) and 24 h post-Dox treatment (b) cells by RT-qPCR. The indicated gene expression was normalized to the level of 18S rRNA and siCON was set as 1. **(c, d and e)** iSLK cells were infected with KSHV virions produced from BAC16 iSLK cells at 48 h post indicated siRNA transfection. (c) Western blot analysis of RIG-I, MDA5 and MAVS in cell lysate from 24 h post-infection iSLK cells. GAPDH was blotted as loading control. (d) Quantification of KSHV infected GFP positive cells by flow cytometry. (e) RNA was extracted from cells and expression of LANA was quantified 24 h post-infection by RT-qPCR. The indicated gene expression was normalized to the level of 18S rRNA and siCON was set as 1. Error bars in all panels represent mean ± SD from three independent experiments. p-values were determined by the Student's t-test, * $p<0.05$, ** $p<0.01$.

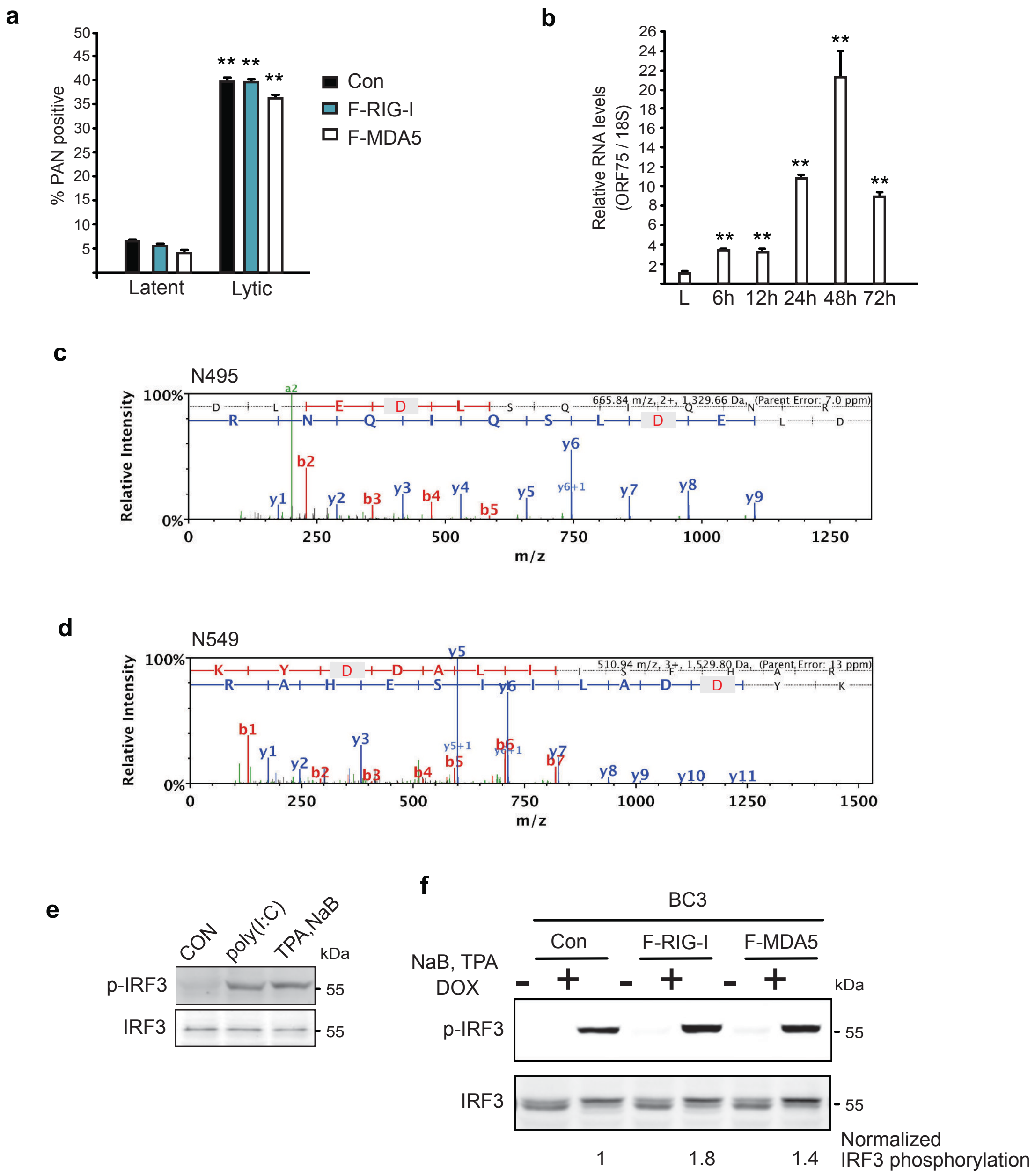

Supplementary Figure 2. BC-3 reactivation induces IRF3 phosphorylation and RIG-I deamidation. **(a)** BC-3 cells were transduced with lentivirus harboring Dox-inducible FLAG tagged RIG-I (F-RIG-I), MDA5 (F-MDA5), or a control empty cassette (Con). The inducible cell lines were treated with Dox, NaB and TPA for 48 h. Lytic reactivation was quantified by performing PAN RNA FISH-Flow. **(b)** BC-3 cells were reactivated with NaB and TPA cells were collected at indicated time point. RNA was extracted from cells and expression of ORF75 was quantified by RT-qPCR. ORF75 expression was normalized to the level of 18S rRNA and latency was set as 1. Error bars in all panels represent mean ± SD from three independent experiments. p-values were determined by the Student's t-test, * $p<0.05$, ** $p<0.01$. **(c and d)** F-RIG-I immunoprecipitation was conducted on lytic reactivated inducible F-RIG-I BC-3 cells and subjected to mass spectrometry. Mass spectrum demonstrating deamidation of D495 and D549 in (c) and (d), respectively. **(e)** BC-3 cells were either transfected with poly(I:C) or treated with NaB and TPA. Total and phosphorylated IRF3 were detected by Western blot in cells harvested 12 h post treatment. **(f)** Cell lysates from latent and 24 h post-reactivation samples were immunoblotted for total and phosphorylated IRF3.

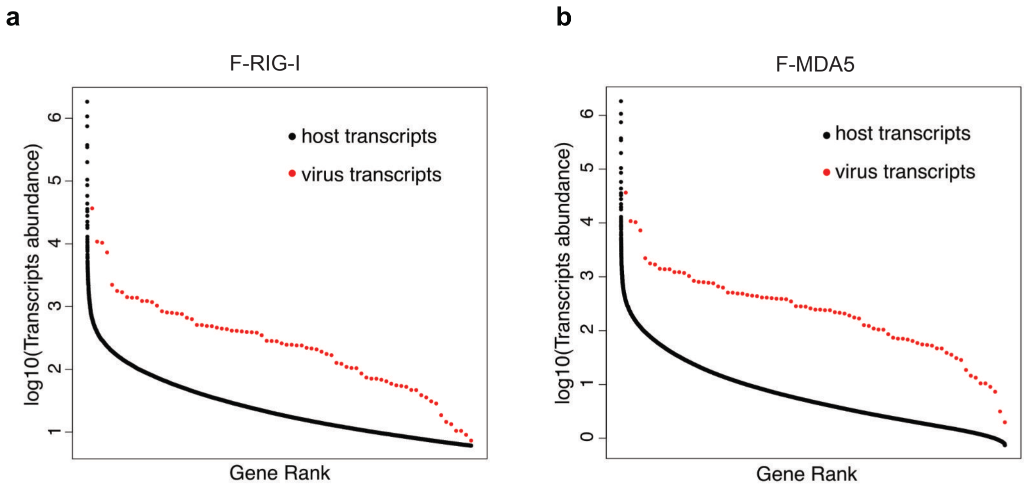

Supplementary Figure 3. Read counts of KSHV- and host-derived transcripts in input RNA-sequencing libraries. (a) Lytic BC-3 cells expressing F-RIG-I. **(b)** Lytic BC-3 cells expressing F-MDA5.

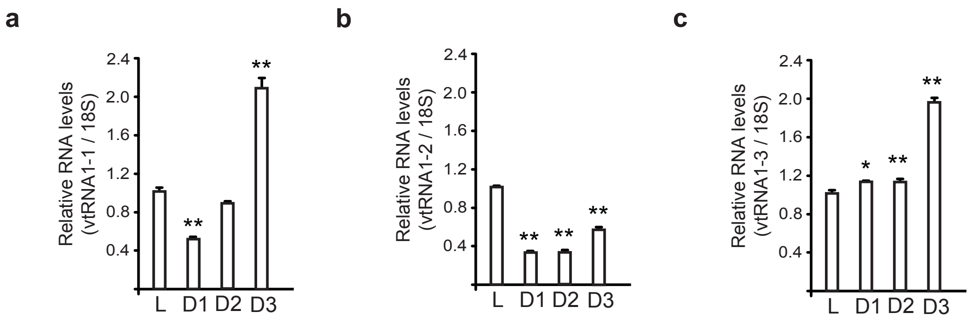

Supplementary Figure 4. Expression of vtRNAs during BC-3 lytic reactivation. BC-3 cells were reactivated for 3 days. (a) vtRNA1-1. **(b)** vtRNA1-2. **(c)** vtRNA1-3. Expression of vtRNAs were quantified by RT-qPCR and their levels were normalized to 18S rRNA. L, latency; D1 to D3, lytic reactivated for 1 day to 3 days. The latent sample was set as 1. The error bars in all panels represent mean ± SD from three independent experiments, and the p-values were determined by the Student's t-test, * $p<0.05$, ** $p<0.01$.

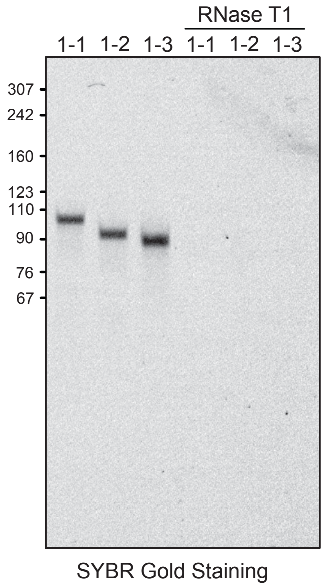

Supplementary Figure 5. In vitro transcribed vtRNAs are not bound to a complementary RNA. SYBR-Gold staining of in vitro transcribed vtRNAs with or without RNase T1 treatment.

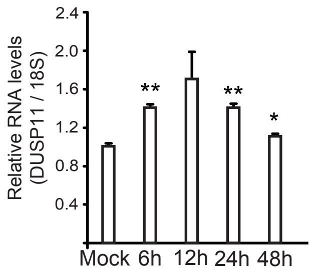

Supplementary Figure 6. DUSP11 expression during *de novo* infection. iSLK cells were infected with KSHV virions and RNA harvested at indicated time point and DUSP11 was quantified by RT-qPCR. Mock indicated no virus infection. DUSP11 gene expression was normalized to the level of 18S rRNA and Mock was set as 1. Error bars in all panels represent mean ± SD from three independent experiments. p-values were determined by the Student’s t-test, * $p<0.05$, ** $p<0.01$.

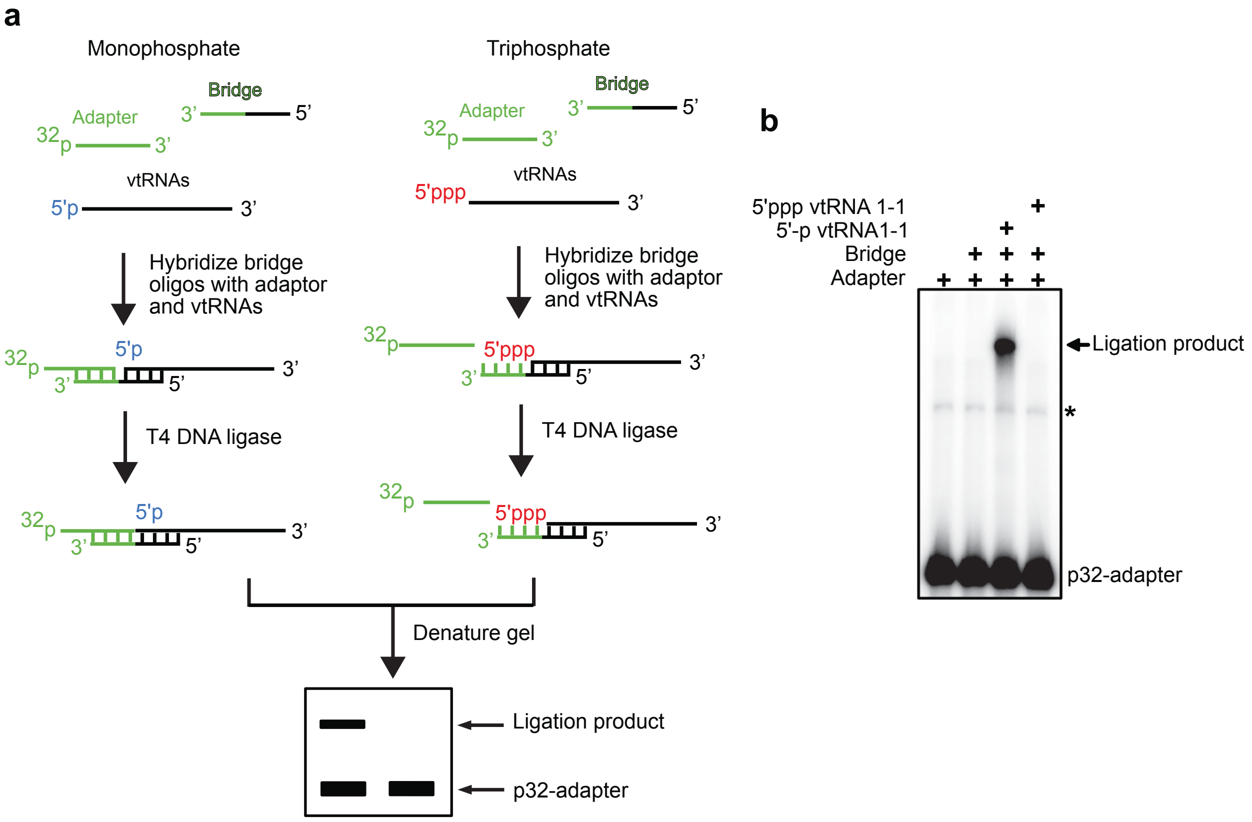

Supplementary Figure 7. Splint-ligation discriminates 5'-phosphate status of RNA. (a) Schematic of splint-ligation assay. A P^{32}-labeled adapter oligonucleotide and bridge oligonucleotide, which base-pairs to both the adapter and vtRNA is annealed and ligated using T4 DNA ligase. Samples are then resolved by urea PAGE gel and visualized by autoradiography. **(b)** In vitro transcribed vtRNA 1-1, which contains a triphosphate, or CIP-treated and subsequently monophosphorylated vtRNA 1-1 were used in splint-ligation assays as described in (a). An adapter, and adapter plus bridge oligonucleotide were ran as controls. *, denotes an adapter-adapter ligation product as it is present in the adapter only lane.

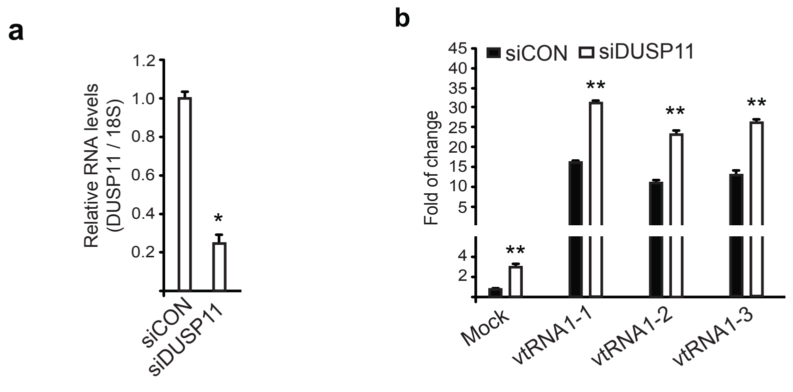

Supplementary Figure 8. Depletion of DUSP11 elicits an interferon response. (a) Quantification of DUSP11 knockdown in HCT116 ISG54-luciferase reporter cell line by RT-qPCR. **(b)** vtRNAs were transfected to CON or DUSP11 depleted HCT116 ISG54-luciferase reporter cells. Cells were harvested 12 h post transfection and subjected to luciferase assay. Mock indicated cells without RNA transfection and siCON Mock was set as 1. The fold of change was normalized to siCON Mock. Error bars in all panels represent mean ± SD from three independent experiments. p-values were determined by the Student's t-test, * $p<0.05$, ** $p<0.01$.

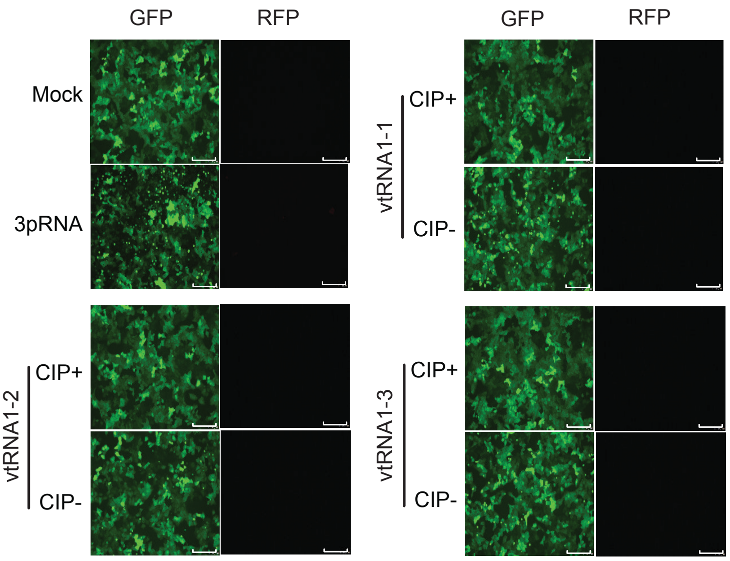

Supplementary Figure 9. Transfection of vtRNAs does not promote KSHV spontaneous lytic reactivation in iSLK.219 cells. iSLK.219 cells were mock transfected, or transfected with 100 ng in vitro transcribed vtRNAs with or without CIP treatment, or a RIG-I ligand RNA (3pRNA). GFP and RFP images were captured 48 h post transfection. Bar indicates 300 μm.

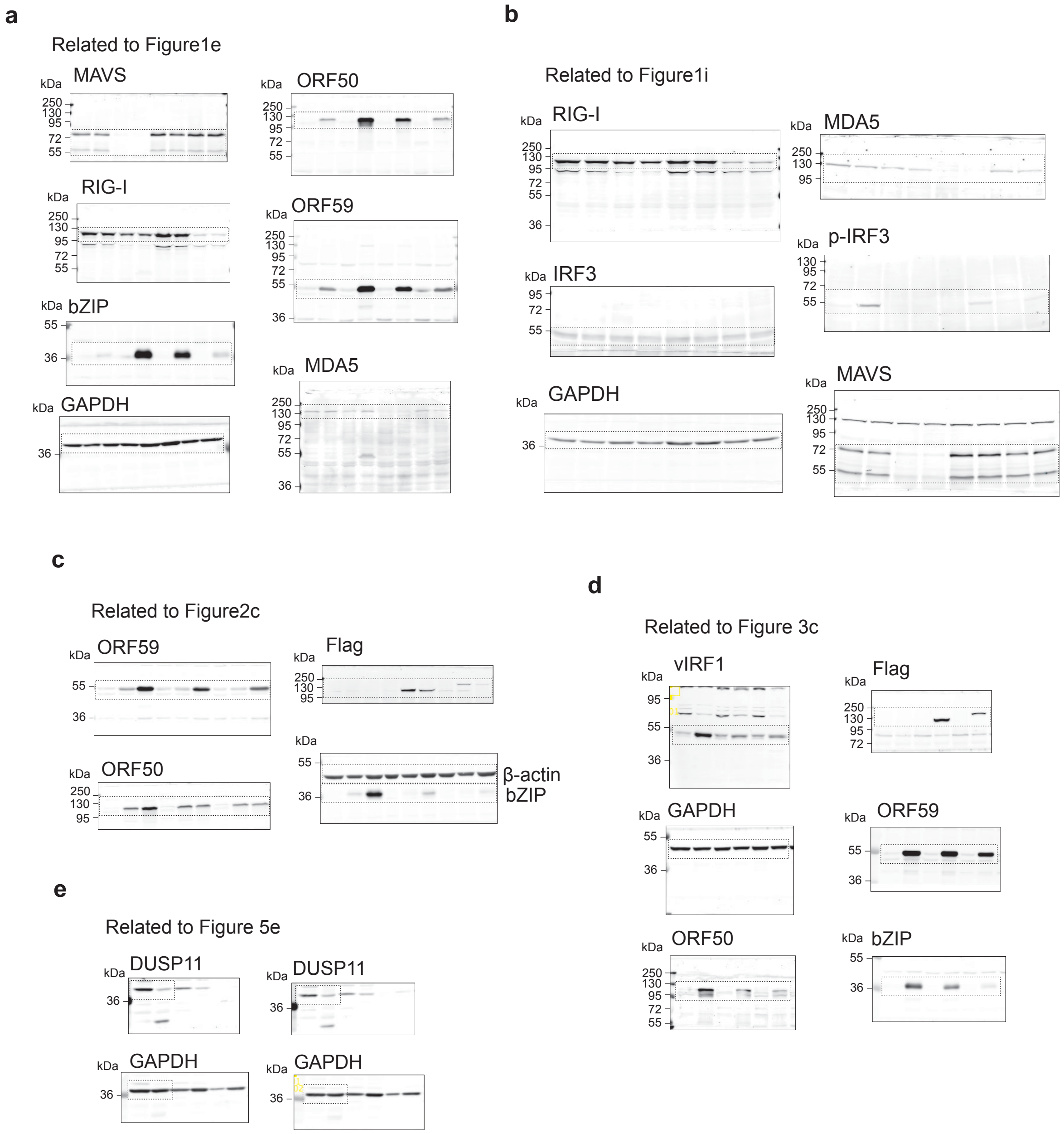

Supplementary Figure 10. Full-size immunoblots and gel scans. (a) Figure 1e. **(b)** Figure 1i. **(c)** Figure 2c. **(d)** Figure 3c. **(e)** Figure 5e.

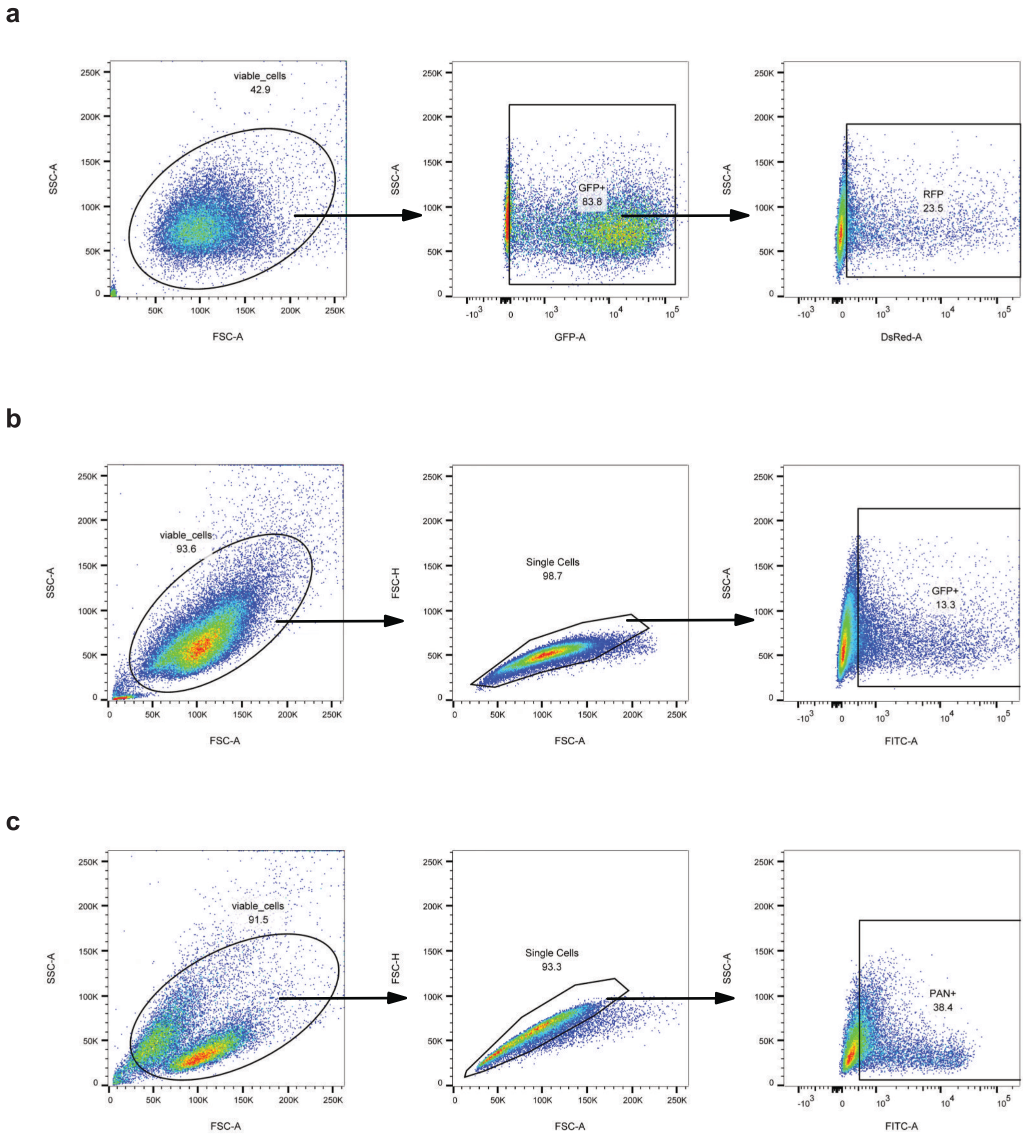

Supplementary Figure 11. Gating strategy used in flow cytometry analysis. (a) Related to Figure 1c. **(b)** Related to Supplementary Figure 1d. **(c)** Related to Supplementary Figure 2a.

Supplementary Table 1. Sequences of oligonucleotide primers and siRNAs.

oligos / siRNA	Sequences
ORF57 F	5’ TGGACATTATGAAGGGCATCCTA
ORF57 R	5’ CGGGTTCGGACAATTGCT
PAN F	5' ATAGGCGACAAAGTGAGGTGGCAT
PAN R	5' TAACATTGAAAGAGCGCTCCCAGC
ORF52 F	5’ AAATCGAAGCCAGGGTCAGG
ORF52 R	5’ CTCCTCTTCGTCGCCTGTTATTG
vIL6 F	5’ CTGTTACCGTACCGGCATCT
vIL6 R	5’ AGAAGCTCCATGACGTCCAC
ORF50 F	5' GAGTCCGGCACACTGTACC
ORF50 R	5' AAACTGCCTGGGAAGTTAACG
ORF59 F	5’ ACAGTCACCGTTTGGTCCTC
ORF59 R	5’ TGTACTCGACGCTGGCATAG
K8 F	5' ATTTCGCAACAGCTTCCAAC
K8 R	5' TACCTGCTGCAGCTGTCTTG
ORF75 F	5' CAGGTCCTTCCTGTGCATGT
ORF75 R	5' GGACAAGGCAACAACGTCAC
LANA F	5' TTGGATCTCGTCTTCCATCC
LANA R	5' ACCAGACGATGACCCACAAC
MAVS F	5' TTGTAGAGATTCTGCCTTACCTG
MAVS R	5' AGGGTATTGAAGAGATGCCA
DDX58 F	5' ATCCCGTTGATCTCCAGGGAA
DDX58 R	5' AGTCTGACTGTCCTTTCTACTTGAAA
IFIH1 F	5' GGGGCATGGAGAATAACTCA
IFIH1 R	5' TGCCCATGTTGCTGTTATGT
vtRNA1-1 F	5' CGACAGTTCTTTAATTGAAACAAGC
vtRNA1-1 R	5' AAGGACTGGAGAGCGCCC
vtRNA1-2 F	5' CTTCGAGTACATTGTAACCACCTC
vtRNA1-2 R	5' AGAGCTGGAAAGCACCCGC
vtRNA1-3 F	5' TTCGCGTGTCATCAAACCACCTC
vtRNA1-3 R	5' AAGAGGGCTGGAGAGCGCC
ISG15 F	5' GCGAACTCATCTTTGCCAGTA
ISG15 R	5' CCAGCATCTTCACCGTCAG
IFI44 F	5' TGGTACATGTGGCTTTGCTC
IFI44 R	5' CCACCGAGATGTCAGAAAGAG
IFIT2 F	5' AAATGCCATTTCACCTGGAACTTG
IFIT2 R	5' GCTTTGAATTCACGATTCTGAAAC
18S rRNA F	5’ GTAACCCGTTGAACCCCATT
18SrRNA R	5’ CCATCCAATCGGTAGTAGCG
NOP14 F	5' GTCAGAGCACACTGACCGAA
NOP14 R	5' CCTCGCCAGGAACTGATTGT
GINS F	5' CAACTGCCTGCCTTCAACGA
GINS R	5' TGCATTTGGCAAGACGCTAC
DUSP11 F	5' GTCTCATCATCAGGCACTTGATGTCC
DUSP11 R	5' CTTGCTCCAGAAGAATGCTTTTCCC
DUSP11 promoter F	5' AGCACAGGTCAATAAGGGCG
DUSP11 promoter R	5' GAACACACAAGGAACCGGGG
DUSP11 TSS F	5' TCATGTGGGTCCCAAGAAGC
DUSP11 TSS R	5' GGGTGCATACACGCCCTTAT
DUSP11 +1.8kb F	5' AATGAAACGAGTCCCAGGCA
DUSP11 +1.8kb R	5' AGAGAGGACATGTTGTGCGG
T7-VTRNA1-1 F	5'<br>TAATACGACTCACTATAGGGCTGGCTTTAGCTCAGCGGTTACTTCGACAGTTCTTTAATTG<br>AAACAAGCAACCTGTCTGGGTTGTTCGAGACCCGCGGGCGCTCTCCAGTCCTTTT
T7-VTRNA1-1 R	5'<br>AAAAGGACTGGAGAGCGCCCGCGGGTCTCGAACAACCCAGACAGGTTGCTTGTTTCAA<br>TTAAAGAACTGTCGAAGTAACCGCTGAGCTAAAGCCAGCCCTATAGTGAGTCGTATTA
T7-VTRNA1-2 F	5'<br>TAATACGACTCACTATAGGGCTGGCTTTAGCTCAGCGGTTACTTCGAGTACATTGTAACC<br>ACCTCTCTGGGTGGTTCGAGACCCGCGGGTGCTTTCCAGCTCTTTT
T7-VTRNA1-2 R	5'<br>AAAAGAGCTGGAAAGCACCCGCGGGTCTCGAACCACCCAGAGAGGTGGTTACAATGTA<br>CTCGAAGTAACCGCTGAGCTAAAGCCAGCCCTATAGTGAGTCGTATTA
T7-VTRNA1-3 F	5'<br>TAATACGACTCACTATAGGGCTGGCTTTAGCTCAGCGGTTACTTCGCGTGTCATCAAACC<br>ACCTCTCTGGGTTGTTCGAGACCCGCGGGCGCTCTCCAGCCCTCTTTT
T7-VTRNA1-3 R	5'<br>AAAAGAGGGCTGGAGAGCGCCCGCGGGTCTCGAACAACCCAGAGAGGTGGTTTGATG<br>ACACGCGAAGTAACCGCTGAGCTAAAGCCAGCCCTATAGTGAGTCGTATTA
vtRNA bridge	5' /5SpC3/CCGCTGAGCTAAAGCCAGCCCTCGCACGTGCCTAGCTAGC/3SpC3/
vtRNA_splint_adapter	5' GTCAGCTAGCTAGCTGCGCTAAGCTTAGCTTAGCTAGCTAGGCACGTGCGAG
vtRNA_All_TEG_Biotin-1	5' CCGCTGAGCTAAAGCCAGCC/3BioTEG/
vtRNA_All_TEG_Biotin-2	5' TGGAGAGCGCCCGCGGGTCT/3BioTEG/
U1_TEG_Biotin-1	5' CTTCGTGATCATGGTATCTC/3BioTEG/
U1_TEG_Biotin-2	5' GAGTGCAATGGATAAGCCTC/3BioTEG/
U1_TEG_Biotin-3	5' CCCCACTACCACAAATTATG/3BioTEG/
siMAVS	5'-rCrCrArCrCUUrGrAUrGrCrCUrGUrGrArATT
siDDX58	5'-rArAUUrCrAUrCrArGrArGrAUrArGUrCrAtt
siIFIH1	5'-rGUUrAUrArGUUrCUUrGUrCrArAUrAtt
siDUSP11-1	5'-rGrArArArCUrGUUrCrCUUrArCUUrArATT
