# Supplementary material for: RIG-I like receptor sensing of host RNAs facilitates the cell-intrinsic immune response to KSHV infection
Source: Nat Commun. 2018 Nov 19;9:4841. doi: 10.1038/s41467-018-07314-7 (PMC6242832; doi:10.1038/s41467-018-07314-7)
Supplement: Supplementary file 3 — Reporting Summary [file 41467_2018_7314_MOESM3_ESM.pdf]

nature research

Corresponding author(s): John Karijolich

Reporting Summary

Nature Research wishes to improve the reproducibility of the work that we publish. This form provides structure for consistency and transparency in reporting. For further information on Nature Research policies, see Authors & Referees and the Editorial Policy Checklist.

Statistical parameters

When statistical analyses are reported, confirm that the following items are present in the relevant location (e.g. figure legend, table legend, main text, or Methods section).

n/a	Confirmed	
☐	☒	The exact sample size (n) for each experimental group/condition, given as a discrete number and unit of measurement
☐	☒	An indication of whether measurements were taken from distinct samples or whether the same sample was measured repeatedly
☐	☒	The statistical test(s) used AND whether they are one- or two-sided<br>*Only common tests should be described solely by name; describe more complex techniques in the Methods section.*
☒	☐	A description of all covariates tested
☐	☒	A description of any assumptions or corrections, such as tests of normality and adjustment for multiple comparisons
☐	☒	A full description of the statistics including central tendency (e.g. means) or other basic estimates (e.g. regression coefficient) AND variation (e.g. standard deviation) or associated estimates of uncertainty (e.g. confidence intervals)
☒	☐	For null hypothesis testing, the test statistic (e.g. F, t, r) with confidence intervals, effect sizes, degrees of freedom and P value noted<br>*Give P values as exact values whenever suitable.*
☒	☐	For Bayesian analysis, information on the choice of priors and Markov chain Monte Carlo settings
☒	☐	For hierarchical and complex designs, identification of the appropriate level for tests and full reporting of outcomes
☒	☐	Estimates of effect sizes (e.g. Cohen's d, Pearson's r), indicating how they were calculated
☐	☒	Clearly defined error bars<br>*State explicitly what error bars represent (e.g. SD, SE, CI)*

Our web collection on statistics for biologists may be useful.

Software and code

Policy information about availability of computer code

Data collection	No software was used.
Data analysis	fastq files were accessed by FastQC (www.bioinformatics. babraham.ac.uk/projects/fastqc). Reads were aligned to the human reference genome (gencode GRCh38.p10) and KSHV genome (GQ994935.1) using Spliced Transcripts Alignment to a Reference (STAR) software. Transcript and gene abundances, as well as depletion/enrichment significance was determined using cufflinks and cuffdiff 2. Statistical analyses and virtualization were performed using R packages: principle component analysis was performed using the PCA function in package FactoMineR 46 and presented with factoextra, genome coverages were presented with Gviz. The gene ontology overrepresentation analysis was performed with clusterprofiler on GO biological process.

For manuscripts utilizing custom algorithms or software that are central to the research but not yet described in published literature, software must be made available to editors/reviewers upon request. We strongly encourage code deposition in a community repository (e.g. GitHub). See the Nature Research guidelines for submitting code & software for further information.

Data

Policy information about availability of data

All manuscripts must include a data availability statement. This statement should provide the following information, where applicable:

- Accession codes, unique identifiers, or web links for publicly available datasets
- A list of figures that have associated raw data
- A description of any restrictions on data availability

Sequencing data from this study have been deposited in GEO under accession number GSE116650.

Field-specific reporting

Please select the best fit for your research. If you are not sure, read the appropriate sections before making your selection.

☒ Life sciences ☐ Behavioural & social sciences ☐ Ecological, evolutionary & environmental sciences

For a reference copy of the document with all sections, see nature.com/authors/policies/ReportingSummary-flat.pdf

Life sciences study design

All studies must disclose on these points even when the disclosure is negative.

Sample size	No statistical methods were used to determine sample size. The number of biological replicates are indicated in the figure legends.
Data exclusions	No data were excluded from the analysis.
Replication	All replication attempts were successful.
Randomization	No randomization done for these experiments.
Blinding	Personnel handling the sequencing of fRIP-seq data were blinded.

Reporting for specific materials, systems and methods

Materials & experimental systems

n/a	Involved in the study	
☒	☐	Unique biological materials
☐	☒	Antibodies
☐	☒	Eukaryotic cell lines
☒	☐	Palaeontology
☒	☐	Animals and other organisms
☒	☐	Human research participants

Methods

n/a	Involved in the study	
☒	☐	ChIP-seq
☐	☒	Flow cytometry
☒	☐	MRI-based neuroimaging

Antibodies

Antibodies used	GAPDH (Invitrogen, GA1R, #MA5-15738), β-actin (Invitrogen, BA3R, #MA5-15739), vIRF1 (Dr. Gary Hayward, John Hopkins University), ORF50 and ORF59 (Dr. Britt Glaunsigner, UC Berkeley), ORF57 (Dr. Zhi-Ming Zheng, National Cancer Institute), bZIP (Dr. Cyprian Rossetto, University of Nevada, Reno), IRF3 (Cell Signaling Technology, D83B9, #4302), Phospho-IRF3 S386 (Abcam, EPR2346, #ab76493), FLAG (Thermo Fisher Scientific, FG4R, MA1-91878), MAVS (Bethyl, #A300-782A), DDX58 (Abcam, EPR18629, #ab180675), MDA5 (Cell Signaling Technology,D74E4, #5321), DUSP11 (Proteintech, #10204-2-AP), AlexaFluor 680-conjugated secondary antibody goat anti-rabbit (Life Technologies, #A27042), AlexaFluor 680-conjugated secondary antibody goat anti-mouse (Life Technologies, #A28183).
Validation	GAPDH (Invitrogen, GA1R, #MA5-15738) and β-actin (Invitrogen, BA3R, #MA5-15739) are validated on manufacturer website for western blot.<br><br>IRF3 (Cell Signaling Technology, D83B9, #4302) validated on manufacturer website for western blot.<br><br>Phospho-IRF3 S386 (Abcam, EPR2346,#ab76493) validated on manufacturer website for western blot.

FLAG (Thermo Fisher Scientific, FG4R, MA1-91878) validated on manufacturer website for western blot. We also validated this antibody in cells not expressing FLAG-tagged protein.

AlexaFluor 680-conjugated secondary antibody (Life Technologies #A27042, #A28183) validated for western blot on manufacturer website.

DDX58 (Abcam, EPR18629, #ab180675) validated on manufacturer website for western blot. We also validated this antibody in DDX58 knockdown cells.

MAVS (Bethyl, #A300-782A) validated on manufacturer website for western blot. We also validated this antibody in MAVS knockdown cells.

MDA5 (Cell Signaling Technology, D74E4, #5321) validated on manufacturer website for western blot. We also validated this antibody in MDA5 knockdown cells.

DUSP11 (Proteintech, #10204-2-AP) validated on manufacturer website for western blot. We also validated this antibody in DUSP11 knockdown cells.

Eukaryotic cell lines

Policy information about cell lines

Cell line source(s)	BC-3 and iSLK.219 cells were provided by Dr. Britt Glaunsinger (UC Berkeley), HEK293T cells were purchased from ATCC.
Authentication	Cell lines were obtained from investigators who generated them, and then passed through an intermediary, or generated in our lab. They are not further authenticated.
Mycoplasma contamination	All cell lines used in this study were mycoplasma free. Cell lines were routinely tested for mycoplasma by PCR.
Commonly misidentified lines (See ICLAC register)	None

Flow Cytometry

Plots

Confirm that:

☒ The axis labels state the marker and fluorochrome used (e.g. CD4-FITC).

☒ The axis scales are clearly visible. Include numbers along axes only for bottom left plot of group (a 'group' is an analysis of identical markers).

☒ All plots are contour plots with outliers or pseudocolor plots.

☒ A numerical value for number of cells or percentage (with statistics) is provided.

Methodology

Sample preparation	iSLK, iSLK.219 and its derivative cells were fixed with 2% paraformaldehyde. BC-3 and derivative cells were fixed in 4% (vol/vol) paraformaldehyde and permeablized with 1X PBS containing 0.2% (vol/vol) Tween-20. The permeabilized cells were then hybridized with Fluorescein-12-dUTP labeled PAN anti-sense oligos in HB 10% dx buffer (10% (wt/vol) dextran sulfate, 2× saline-sodium citrate (SSC), 10% (vol/vol) formamide, 1 mg/ml tRNA and 0.2 mg/ml BSA) at 37 °C overnight. After extensive washing with HBW buffer (2× SSC, 10% (vol/vol) formamide and 0.2 mg/ml RNase-free BSA), cells were analyzed.
Instrument	iSLK.219 cell flow cytometry analysis was performed on a BD LSR Fortessa instrument and iSLK cell flow cytometry analysis and BC-3 cell FISH-FLOW was performed on a BD Canto II instrument.
Software	FlowJo X software
Cell population abundance	Not applicable
Gating strategy	For iSLK.219 cells we first gated FSC-A and SSC-A. After that, we gated on GFP positive cells and then gated on RFP positive cells. For iSLK cells, we first gated FSC-A and SSC-A. After that, we gated on single cells and then gated on GFP positive cells. For BC-3 cells, we first gated FSC-A and SSC-A. After that, we gated on single cells and then gated on FITC.

☒ Tick this box to confirm that a figure exemplifying the gating strategy is provided in the Supplementary Information.
